# Cyclic Voltammetry of Auranofin

**DOI:** 10.1155/MBD.1999.233

**Published:** 1999

**Authors:** Ahmed A. Mohamed, Alice E. Bruce, Mitchell R. M. Bruce

**Affiliations:** Department of Chemistry University of Maine Orono Maine 04469-5706 USA

## Abstract

The oxidative behavior of Auranofin, 2,3,4,6-tetra-O-acetyl-1-thio-β-D-glucopyranosato- S(triethylphosphine)gold(I), was investigated by using cyclic voltammetry (CV) in 0.1 M Bu_4_NPF_6_/CH_2_Cl_2_ and 0.1 M Bu_4_NPF_4_/CH_2_Cl_2_ solutions using Pt working and auxiliary electrodes and a Ag/AgCI reference. CV studies at scan rates from 50-2,000 mV/s and Auranofin concentrations between 1 and 4 mM, show two irreversible oxidation processes occurring at +1.1 V and +1.6 V vs. Ag/AgCl. Ph_3_ (*p*-thiocresolate) was also investigated as a reference for comparison of the oxidation processes in Auranofin to that of other phosphine gold thiolate complexes previously reported. The electrochemical response appears to be sensitive to adsorption at the electrode as well as to the nature of the supporting electrolyte solution. Repeated cycling shows a build up of products at the electrode.


### CYCLIC VOLTAMMETRY OF AURANOFIN

Ahmed A. Mohamed, Alice E. Bruce,* and Mitchell R. M. Bruce*

Department of Chemistry, University of Maine, Orono, Maine 04469-5706, USA

### ABSTRACT

The oxidative behavior of Auranofin, 2,3,4,6-tetra-O-acetyl-1-thio-β-D-glucopyranosato-S(triethylphosphine)gold(I), was investigated by using cyclic voltammetry (CV) in 0.1 M $Bu_4NPF_6/CH_2Cl_2$ and 0.1 M $Bu_4NBF_4/CH_2Cl_2$ solutions using Pt working and auxiliary electrodes and a Ag/AgCl reference. CV studies at scan rates from 50-2,000 mV/s and Auranofin concentrations between 1 and 4 mM, show two irreversible oxidation processes occurring at +1.1 V and +1.6 V *vs.* Ag/AgCl. $Ph_3PAu$(*p*-thiocresolate) was also investigated as a reference for comparison of the oxidation processes in Auranofin to that of other phosphine gold thiolate complexes previously reported. The electrochemical response appears to be sensitive to adsorption at the electrode as well as to the nature of the supporting electrolyte solution. Repeated cycling shows a build up of products at the electrode.

### INTRODUCTION

The medicinal effects of gold drugs have been extensively investigated during the last two decades.[1] However, a recent review of the electrochemical literature shows that the redox data for biologically important gold and silver complexes is generally still lacking.[2] This is especially significant for gold drugs, where the redox reactivity, especially oxidation, has been invoked in partial explanation of both therapeutic and toxic side effects. Auranofin, (2,3,4,6-tetra-O-acetyl-1-thio-β-D-glucopyranosato-S)(triethylphosphine)gold(I), is a water insoluble phosphine gold thiolate complex that is used as an orally active anti-arthritic drug in both experimental animals and man. Reductive polarography of Auranofin has previously been reported.[3] We now report oxidative cyclic voltammetry studies of Auranofin.

### MATERIALS AND METHODS

**Materials.** Methylene chloride and the supporting electrolytes, tetra-N-butylammonium hexafluorophosphate ($Bu_4NPF_6$) and tetra-N-butylammonium tetrafluoroborate ($Bu_4NBF_4$) were used as received (Aldrich). Auranofin was purchased from Pfanstiehl Laboratories, Inc., IL. $Ph_3PAu$(*p*-tc) was prepared according to previously published methods.[4]

**Abbreviations.** The following abbreviations are used: *p*-tc = *p*-thiocresol, pdt = propane dithiolate, dppe = diphenylphosphine ethane, rev. = reversible, irr. = irreversible.

**Cyclic Voltammetry (CV) Experiments.** CV experiments were conducted using an EG&G Princeton Applied Research 273 potentiostat/galvanostat under computer control. CV measurements were performed in methylene chloride with 0.1 M $Bu_4NPF_6$ or 0.1 M $Bu_4NBF_4$ as supporting electrolyte. Fresh solutions containing electrolyte (10 ml) were prepared prior to each CV experiment. Each solution was deoxygenated by purging with nitrogen for 2-5 minutes. Background CV's were acquired before the addition of gold complex. A three-electrode system was used, comprised of a platinum (1.6 mm diameter) working electrode, a platinum wire auxiliary electrode, and a silver/silver chloride (Ag/AgCl) reference electrode. The working electrode was wiped prior to each experiment. The auxiliary electrode was lightly sanded before each set of experiments with fine sandpaper. Potentials are reported vs. Ag/AgCl at room temperature and are not corrected for junction potentials. Each CV experiment was repeated a number of times.

### RESULTS

The results of cyclic voltammetry experiments on Auranofin are shown in Figure 1. Figure 1 a-b are the current-voltage responses for 1 mM Auranofin in 0.1 M $Bu_4NBF_4/CH_2Cl_2$ solutions at scan rates of 50 mV/s (a) and 500 mV/s (b), respectively. In each CV, there are two anodic processes. In Figure 1a they occur at about +1.1 V (*vs.* Ag/AgCl) and +1.6 V, while in Figure 1b, they occur at somewhat higher potentials. The two redox processes were found to be irreversible at all scan rates, concentrations, and switching potentials (e.g. +1.2 V) investigated. Both processes appear characteristic of an EC mechanism, with the following reaction being quite fast. CV experiments with ferrocene, a reversible one-electron redox couple, at 50 mV/s and 500 mV/s show a potential shift of similar magnitude as seen for Auranofin (Figure 1a-b). This result suggests that the shift originates from the electrochemical cell used, such as from cell resistance (*i*R drop), and not from kinetic effects of a following reaction.

Figure 1 c-e, shows the effect of change of concentration as well as of electrolyte. The current-voltage response for 1 mM Auranofin in 0.1 M $Bu_4NPF_6/CH_2Cl_2$ solution at scan rates of 50 mV/s is shown in Figure 1c. This CV is the result of the same experimental procedure used to generate the CV for Figure 1a, except for a change of electrolyte ($Bu_4NPF_6$ *vs.* $Bu_4NBF_4$). At first glance, the CV wave-shapes of Figure 1c *vs.* 1a appear significantly different. However, close inspection of Figure 1c shows similar anodic processes to 1a, i.e. one starting at about +1.0 V as well as one at +1.65 V. What is also apparent is the great diminution of the current response of Figure 1c compared to Figure 1a. It has been previously demonstrated that the CV wave-shapes for phosphine gold thiolate complexes are sensitive to the type of electrode as well as to solvent owing to the effects of adsorption at the electrode.[5] Repeated CV cycling of Auranofin also leads to filming of the electrode, indicative of adsorption at the electrode. Presumably, the filming process is affected during oxidation by the rate at which oxidized (positively charged) complexes are removed from the surface of the electrode. It is therefore not unexpected that changing the anion ($PF_6^-$ *vs.* $BF_4^-$) would also effect the wave-shape. The data suggests that the $PF_6^-$ anion is not as efficient as $BF_4^-$ in keeping the electrode clear of oxidation products, leading to an increase in filming rate and thus leading to a great reduction in the current response. The effect of increasing the concentration of Auranofin from 1 mM, 2 mM, and 4 mM is seen in Figures 1c, 1d, and 1e respectively. The increase in the current response of both oxidation processes demonstrates that the observed electrochemistry originates from the analyte and not the solvent or electrolyte.

Cyclic voltammetry experiments were also performed on $Ph_3PAu$(*p*-thiocresolate) under similar conditions used for Auranofin (0.1 M $Bu_4NBF_4/CH_2Cl_2$ solutions at scan rates of 50 mV/s). The results allow comparison to other electrochemical investigations reported for phosphine gold thiolate complexes.[5] Table 1 summarizes the results from this study and related complexes.

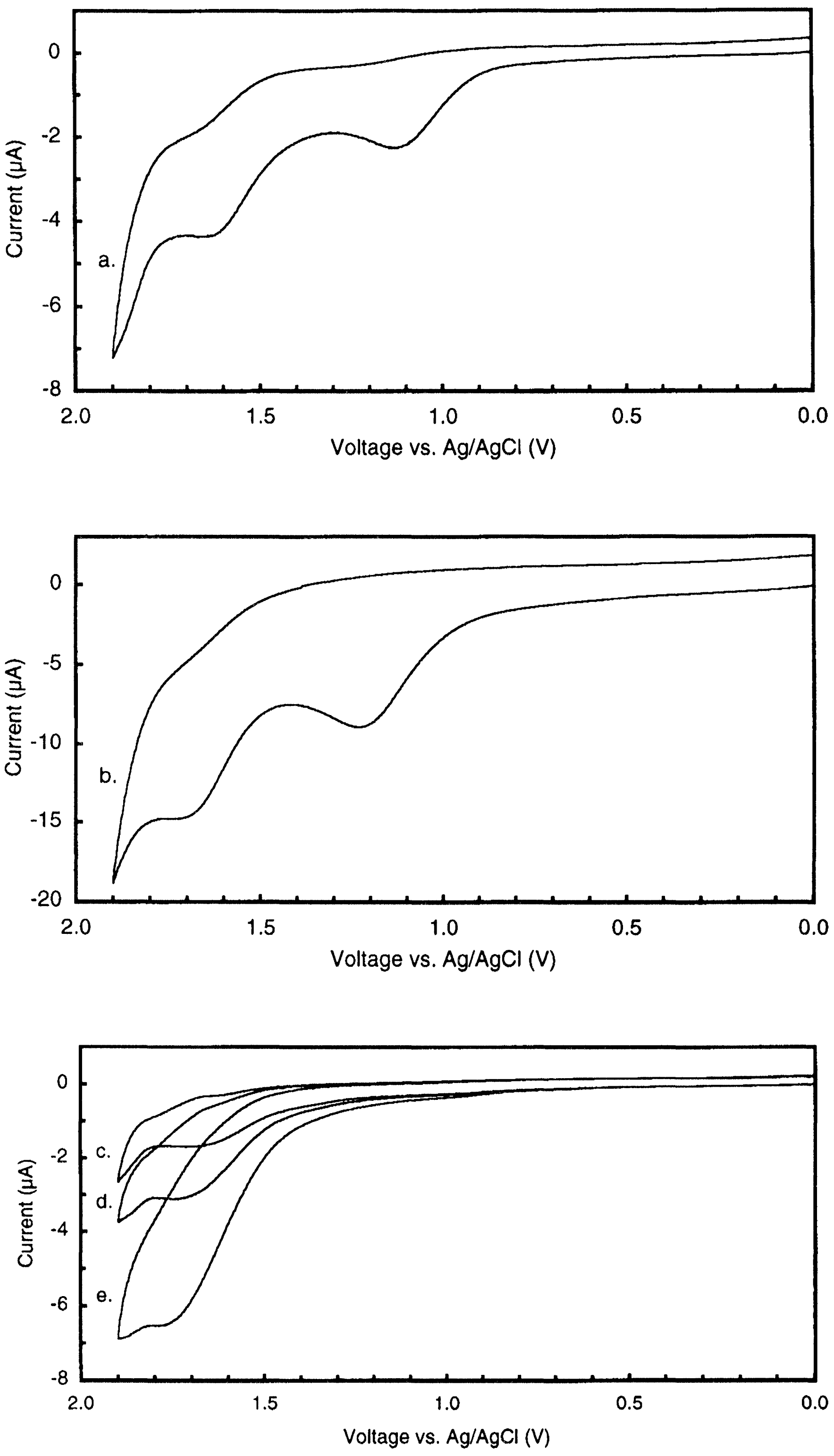

**Figure 1.** Cyclic Voltammograms of Auranofin using Pt working and auxiliary electrodes and Ag/AgCl reference: (a-b) 1 mM Auranofin in 0.1 M $Bu_4NBF_4/CH_2Cl_2$; (a) at 50 mV/s, (b) at 500 mV/s. (c-e) in 0.1 M $Bu_4NPF_6/CH_2Cl_2$ at 50 mV/s; (c) 1 mM, (d) 2 mM, (e) 4 mM.

**Table 1.** Cyclic Voltammetry Data for Auranofin, $Ph_3PAu$(*p*-tc), and Related Complexes.

| Complex | Oxidations | Reductions | Solvent | Ref. Elec. | Ref. |
|---|---|---|---|---|---|
| Auranofin | +1.1 (irr) [a] | | $CH_2Cl_2$ | Ag/AgCl | b |
| | +1.6 (irr) [a] | | $CH_2Cl_2$ | Ag/AgCl | b |
| | | -0.5(rev) [c] | $C_2H_5OH/H_2O$ | SCE | 3 |
| $Ph_3PAu$(*p*-tc) | +0.82 (irr) | | $CH_2Cl_2$ | Ag/AgCl | b |
| | +1.50 (irr) | | $CH_2Cl_2$ | Ag/AgCl | b |
| $Au_2(\mu$-dppe)(*p*-tc)$_2$ | +0.72 (Sulfur) | | $CH_2Cl_2$ | SCE [d] | 5a |
| | +1.54 ($Au^{I/III}$) | | $CH_2Cl_2$ | SCE [d] | 5a |
| $Au_2(\mu$-dppe)(pdt) | +0.77 (Sulfur) | | $CH_2Cl_2$ | SCE [d] | 5a |
| | +1.20 ($Au^{I/III}$) | | $CH_2Cl_2$ | SCE [d] | 5a |
| $Me_3PAu$(*p*-tc) | +0.93 (irr) | | $CH_3CN$ | Ag/AgCl | 5b |
| | +1.55 (irr) | | $CH_3CN$ | Ag/AgCl | 5b |

a. CV experiment at 1 mM Auranofin and 50 mV $s^{-1}$ using 0.1 M $Bu_4NBF_4$ solution. b. This work. c. Reversible at pH > 9; assigned to the $Au^{I/0}$ couple. d. Pt wire working electrode and 0.1 M TBAH solution; scan rate of 50 mV $s^{-1}$.

### DISCUSSION

Auranofin has been investigated by a variety of techniques.[3,6,7] X-ray crystallography shows that Auranofin is monomeric with a nearly linear S-Au-P linkage (173.6$^0$) and virtually identical Au-S (2.29 Å) and Au-P (2.26 Å) bond lengths.[6] The triethylphosphine group appears to stabilize the gold-thiol moiety.[6] The $^{197}$Au Mossbauer for Auranofin shows relatively high parameters (IS = 3.55 mm/sec, QS= 8.64 mm/sec, relative to gold) compared to Myocrisin and Solganol, intrinsic to the Au-P bond.[6] Auranofin is luminescent in the solid state and in EtOH glasses at 77K ($\lambda_{max}$ = 618nm).[7] Auranofin is also photoreactive ($\lambda$ = 254 nm) in acetonitrile, undergoing photodecomposition that appears not to include production of elemental gold.[7]

The dc and differential pulse polarography studies of Auranofin by Perez and coworkers[3] at a dropping mercury electrode, establishes that Auranofin undergoes a diffusion controlled and reversible reductive redox process at –0.5 V (*vs.* SCE) at pH greater than 9.5. Bulk electrolysis at –0.8 V yields an n value of 1 indicative that reduction involves the $Au^{I/0}$ couple. Below a pH of about 8.5, a proton dependent pathway occurs.

Oxidation of Auranofin with hypochlorite, a strong oxidant released by phagocytic cells, has also been studied by Shaw and coworkers.[8] Sulfonate and $Et_3PO$ formed first followed by oxidation of Au(I) to Au(III).

This work reports oxidative cyclic voltammetry studies on Auranofin in a non-aqueous solvent ($CH_2Cl_2$). There are two oxidation processes that occur at +1.1 V and +1.6 V. Both appear to be irreversible at all scan rates investigated (50-2,000 mV/s). Table 1 shows the results of CV studies on Auranofin and related phosphine gold thiolate complexes.[9] All of the complexes show two irreversible oxidation processes that are separated by 400-800 mV. The first oxidation process for the $PPh_3$ and dppe complexes have been assigned as a sulfur based oxidation.[5] Recent results from our laboratory on binuclear as well as on mononuclear phosphine gold thiolate complexes indicate that after an initial one-electron sulfur-based oxidation, the gold complex rapidly undergoes rearrangement to form a gold cluster and disulfide.[10] This suggests the possibility that one-electron oxidation of Auranofin may provide a pathway to higher molecular weight gold complexes as well as a mechanism to increase disulfide concentration. Characterization of the one-electron oxidation products of Auranofin is currently underway in our lab and this information may ultimately be important in explaining the reactivity of gold-sulfur complexes in the body.

### ACKNOWLEDGEMENT

The authors are grateful to J. Chen for synthesis of pure $Ph_3PAu$-*p*-tc. Johnson Matthey is acknowledged for a generous loan of $HAuCl_4$.

### REFERENCES

1. (a) Berners-Price, S. J.; Sadler, P. J. in *Frontiers in Bioinorganic Chemistry*, Xavier, A. V., Ed., VCH, Weinheim, Germany, 376-388 (1986). (b) Berners-Price, S. J.; Sadler, P. J. *Structure and Bonding*, **70**, 27-102 (1988). (c) Sadler, P. J.; Ni Dhubhghaill, O. M. in *Metal Complexes in Cancer Chemotherapy*, Keppler, B. K., Ed., VCH, Weinheim, New York, 222-248 (1993). (d) Brown, D. H. and Smith, W. E. *Chem. Soc. Rev.*, **9**, 217-239 (1980). (e) Parish, R. V.; Cottrill, S. M. *Gold Bull.*, **20**, 3 (1987). (f) Fricker, S. P. *Gold Bull.*, **29**, 53-60 (1996). (g) Shaw III, C. F. *Comments Inorg. Chem.*, **8**, 233-267(1989). (h) Fricker, S. P. *Trans. Met. Chem.*, **21**, 377-383 (1996). (i) *Auranofin in Rheumatoid Arthritis*, Gottlied, N. L., Ed., ADIS press, New Zealand (1987). (j) *Auranofin*, Proceedings of a Smith Kline & French International Symposium, Capell, H. A.; D. S.; Manghani, K. K.; Morris, R. W., Eds., Excerpta Medica, Tokyo(1983). (k) Dash, K. C.; Schmidbaur, H. in *Metal Ions in Biological Systems*, Sigel, H., Ed., Chapter 6, 179-205 (1982). (l) Kean, W. F.; Hart, L.; Buchanan, W. W. *British J. Rheum.*, **36**, 560-572 (1997).
2. Mohamed, A. A.; Bruce, A. E.; Bruce, M. R. M. *Chemistry of Gold and Silver Complexes*, Patai, S., Ed., John Wiley and Sons, in press.
3. Mendez, J. H.; Perez, A. S.; Zamarreno, M. D. *J. Pharm. Sci.*, **78**, 589-591 (1989).
4. Narayanaswamy, R.; Young M. A.; Parkhurst, E.; Ouellette, M.; Kerr, M. E.; Ho, D.; Elder, R. C.; Bruce, A. E.; Bruce, M. R. M. *Inorg. Chem.*, **32**, 2506-2517 (1993).
5. (a) Jiang, T.; Wei, G.; Turmel, C.; Bruce, A. E.; Bruce, M. R. M. *Metal-Based Drugs*, **1**, 419-431. (1994). (b) Jiang, T. *Electrochemical Studies of Gold(I) Phosphine Complexes*, University of Maine, M. Sc. Thesis (1991).
6. Hill, D. T.; Sadler, P. J.; Calis, G.; Trooster, J. M. in *Bioinorganic Chemistry of Gold Coordination Compounds*, Sutton, B.; Franz, R.G., Eds., 67-81 (1983).
7. Kunkely, H.; Vogler, A. *Z. Naturforsch., B: Chem. Sci.* **51**, 1067-1071 (1996).

8. Shaw III, C. F.; Schraa, S.; Gleichmann, E.; Grover, Y. P.; Dunemann, L; Jagarlamudi, A. *Metal-Based Drugs,* **1**, 351-362 (1994).

9. Conversion of SCE to Ag/AgCl reference electrodes can be approximated by adding +0.045 V. For additional information see ref. 2 and references therein.

10 Chen, J.; Bruce, A. E.; Bruce, M. R. M. unpublished results.

**Received: December 14, 1998 -**
**Accepted in final form: February 19, 1999**

